# Management of Foley catheter induction among nulliparous women: a retrospective study

**DOI:** 10.1186/s12884-015-0715-9

**Published:** 2015-10-27

**Authors:** Heidi Kruit, Oskari Heikinheimo, Veli-Matti Ulander, Ansa Aitokallio-Tallberg, Irmeli Nupponen, Jorma Paavonen, Leena Rahkonen

**Affiliations:** Department of Obstetrics and Gynecology, University of Helsinki, Helsinki University Hospital, Haartmaninkatu 2, Helsinki, 00029 HUS Finland; Children’s Hospital, University of Helsinki, Helsinki University Hospital, Helsinki, Finland

**Keywords:** Induction of labour, Foley catheter, Caesarean delivery rate, Nulliparous, Maternal infection, Neonatal infection

## Abstract

**Background:**

Induction of labour is associated with increased risk for caesarean delivery among nulliparous women. The aims of this study were to evaluate the risk factors for caesarean delivery and to investigate the risk of maternal and neonatal infections in nulliparous women undergoing induction of labour by Foley catheter.

**Methods:**

This clinical retrospective study of 432 nulliparous women with singleton pregnancy and intact amniotic membranes at or beyond 37 gestational weeks scheduled for induction of labour by Foley catheter was conducted over the course of one year, between January 2012 and January 2013, in Helsinki University Hospital. The main outcome measures were caesarean section rate and maternal and neonatal infections. Univariate and multivariate logistic regressions were used to estimate relative risks by odds ratios with 95 % confidence intervals.

**Results:**

The caesarean section rate was 39.1 % (*n* = 169). In multivariate regression analysis, the factors associated with caesarean section were the need for oxytocin for labour induction [OR 2.9 (95 % CI 1.8-4.5) *p* < 0.001] and early epidural analgesia [OR 9.9 (95 % CI 2.1-47.5), *p* = 0.004]. The maternal intrapartum infection rate was 6.3 %, and the clinical neonatal infection rate was 2.8 %. In multivariate analysis, gestational diabetes was associated with maternal intrapartum infection [OR 4.3 (95 % CI 1.7-11.0*, p* = 0.002] and early epidural analgesia with neonatal clinical sepsis [OR 10.5 (95 % CI 1.4-76), *p* = 0.02].

**Conclusions:**

Oxytocin induction and early epidural analgesia were associated with caesarean delivery. Gestational diabetes and early epidural analgesia were associated with infectious morbidity. Since the first caesarean delivery has a major impact on subsequent pregnancies, optimising labour induction among nulliparous women is important.

## Background

The rates of induction of labour (IOL) are rising, ranging between 20–30 % in developed countries [1, 2]. Both mechanical and pharmacological methods are used for IOL in women with an unfavourable cervix, and vaginal delivery rates are similar [3]. WHO recommends the use of a balloon catheter for IOL (WHO Recommendations for Induction of Labour, World Health Organization, 2011). However, both IOL methods are associated with induction failure and caesarean delivery [4, 5]. The risk of caesarean section in such cases is high, up to 50 %, especially among nulliparous women [6–8]. We found earlier that nulliparous women with prolonged pregnancy and Foley catheter IOL in our hospital had a high caesarean delivery rate of 37.3 % [9]. The reasons for increased caesarean section rates are unclear, but they may in part relate to the subsequent management of induced labour. In addition, previous studies have shown that duration of labour correlates with the risk of maternal chorionamnionitis and neonatal infections [9, 10]. This highlights the need for additional studies. Thus, we wanted to further analyse the factors that may affect the progress of induced labour. The primary objective of the study was to describe labour outcomes in nulliparous women undergoing IOL by Foley catheter, while the secondary objective was to evaluate risk factors for caesarean delivery and maternal and neonatal infections.

## Methods

This clinical retrospective study of 532 nulliparous women with singleton pregnancy ≥ 37 weeks of gestation undergoing IOL by Foley catheter was conducted in the Department of Obstetrics and Gynaecology at the Helsinki University Central Hospital between January 2012 and January 2013. The study protocol was approved by the Helsinki and Uusimaa Hospital District ethics committee for obstetrics and gynaecology, pediatrics and psychiatry (Nr. 268/13/03/03/2012). Approval to carry out the study was granted by the Hospital district of Helsinki and Uusimaa (§20/12042012). The need to collect an informed consent of the participating women was deemed unnecessary by the ethics committee based on national regulations (Medical Research Act 488/1999, chapter 2 a (23.4.2004/295), section 5 and 10a). The patients were identified from hospital records. Exclusion criteria were twin pregnancy (*n* = 7), breech presentation (*n* = 11), prelabour rupture of membranes (*n* = 23) and sequential use of Foley catheter and prostaglandin for IOL (*n* = 59), leaving a total of 432 women. All women had a Bishop score < 6 at the start of IOL.

A single balloon catheter (Rüsch 2-way Foley, Couvelaire tip, catheter size 22 Ch, Teleflex Medical, Athlone, Ireland) was used. The catheter was introduced into the endocervix and the space between the amniotic membrane and the lower uterine segment. The balloon reservoir was inflated with a median of 40 ml (range 30–60 ml) of saline. Thereafter, the balloon was pulled so that it rested on the internal os. Light traction was applied, and the catheter was taped to the inner thigh.

After expulsion of the balloon, amniotomy was performed if the Bishop score was ≥ 6. If spontaneous expulsion of the Foley catheter did not occur within 24 hours, the balloon was removed. If the cervix remained unripe with Bishop score < 6 after balloon expulsion or removal, induction was continued with intravaginal misoprostol. These (*n* = 59) women were excluded from the final analysis.

Interval times were calculated from the hospital records. The duration of Foley catheter retention was defined as the interval time from balloon insertion to expulsion or removal. The timing of amniotomy varied from immediately after expulsion of the balloon to the next morning, depending on cervical ripeness and delivery unit capacity. Amniotomy was categorised as either immediate (≤2 h from balloon expulsion) or delayed (>12 h from balloon expulsion).

Oxytocin for labour induction was administered in the absence of spontaneous regular contractions. Oxytocin was initiated by discretion of the obstetrician; the timing of its administration varied between 2 and 24 hours after spontaneous rupture of membranes. Oxytocin was also routinely used for labour augmentation if needed, but this study only concentrated on the timing of oxytocin for labour induction. Oxytocin induction was categorised as either immediate (≤3 h from rupture of membranes) or delayed (>12 h from rupture of membranes). Regular contractions were defined as contractions every 3 to 5 minutes with cervical dilation of minimum of 3 cm. The induction-to-delivery interval was defined as the time from insertion of the balloon catheter to delivery.

Data on the study population characteristics, maternal antenatal risk factors, mode of delivery, and maternal and neonatal infections were obtained and collected from antenatal clinic charts and hospital records. The main outcomes were the mode of delivery and maternal and neonatal infections. When there was more than one indication for caesarean section, the primary indications were categorised using the following hierarchy: foetal distress, infection, failure to progress and failed induction. Labour arrest in the first stage of labour was defined as failure to progress despite ruptured membranes and a minimum of 4 hours of adequate uterine activity without cervical change. Failed induction was diagnosed after ruptured membranes and 6–12 hours of oxytocin administration without cervical change [11].

Maternal infections were categorised as intrapartum or postpartum (from delivery to discharge). The criteria for intrapartum infection were maternal fever (≥38 °C) during labour, foetal tachycardia (≥160 bpm), uterine tenderness, purulent amniotic fluid or vaginal discharge and total white cell count > 20 e9/l. At least two of these criteria had to be met in combination with administration of antibiotics. Postpartum infections included endometritis, wound infection and puerperal fever of unknown origin.

Neonatal infections were categorised into blood culture-positive sepsis, clinical sepsis, and suspected sepsis. Neonatal clinical sepsis was defined as a blood culture-negative infection with symptoms and signs consistent with sepsis (such as respiratory distress, apnea, tachycardia, poor perfusion, low blood pressure, fever, hypo- or hyperglycemia, irritability, feeding problems, lethargy and convulsions), abnormal blood values (such as elevated levels of C-reactive protein, leucocytosis or leucopenia, increased neutrophil precursors and thrombocytopenia) and positive response to a minimum of 5 days of antibiotic treatment. The cases defined as suspected sepsis had to have at least one symptom and one abnormal laboratory test value, and a positive response to antibiotic treatment.

We performed a post-hoc power analysis by simulations using R software. All calculations were carried out using the Microsoft Statistical Package for Social Sciences (SPSS Inc., Chicago, IL, USA) for Windows v18.0. Categorical variables were compared with the chi-square test and Fisher’s exact test when appropriate. Data with continuous variables were assessed with a T-test when the data followed a normal distribution and with a Mann–Whitney U test if the data did not follow a normal distribution. We used univariate and multivariate logistic regression to estimate relative risks represented by odds ratios (ORs) with 95 % confidence intervals (CIs). The risk factors (seen in Table 4) of caesarean section, maternal infections and neonatal infections were assessed with logistic regression. In multivariate regression analysis, the time of amniotomy after balloon expulsion and the time of oxytocin administration for IOL after amniotomy were used as continuous variables. A P-value of < 0.05 was considered significant.

## Results

The indications for IOL among 432 nulliparous women are shown in Table 1. The mean gestational age was 41.0 (±1.4) at the start of IOL. The mean age of the women was 30.5 (±4.8) with mean BMI of 24.5 (±5.0). Table 2 shows the characteristics of the labour induction. Women with Bishop score ≤ 3 at the start of IOL were more likely to need oxytocin induction than women with Bishop score > 3 (50 % vs. 37.9 %, *p* = 0.01).

**Table 1:** Main indication for IOL (*n* = 432)

	n	Percent
Post-term pregnancy	266	61.6
Pre-eclampsia/hypertension	56	13
Intrauterine growth restriction	23	5.3
Gestational diabetes	29	6.7
DM I	3	0.6
Oligohydramnion	19	4.4
Obstetric cholestasis	16	3.7
Maternal disease^a^	3	0.6
Foetal disease^b^	4	1
Psychosocial reasons^c^	3	0.6
Decreased foetal movement	6	1.4
LGA	4	0.9

*DM I* diabetes mellitus type 1, *LGA* large for gestational age

^a^Back pain, heart transplantation, difficulty urinating, ulcerative colitis

^b^Heart malformation, Catc-22 h, suspected distress

^c^Maternal exhaustion, fear of labour, maternal preference

**Table 2:** Characteristics of the labour induction

	n	Percent
Bishop score ≤ 3 at start of IOL	209	47.9
Time the Foley catheter was retained, median (range)	666	(1–1647)
Spontaneous rupture of the membranes	25	5.8
Spontaneous contractions before balloon expulsion	60	13.9
Oxytocin administration	402	93.1
Oxytocin needed for induction	188	46.7
Early epidural analgesia^a^	15	3.5
Primary inertia^b^	15	3.5

^a^On maternal request in the absence of regular contractions

^b^No contractions despite oxytocin administration for ≥ 6 hours

Maternal outcomes are shown in Table 3. The overall caesarean section rate was 39.1 % (*n* = 169). The post-hoc power analysis indicated that using a two-sided alpha-level of 5 % we had more than 80 % power to detect an odds-ratio (OR) of 1.9 between gestational diabetes (*n* = 81) and rate of cesarean delivery, and an OR of 4.6 between gestational diabetes and neonatal sepsis. Furthermore, we had over 85 % power to detect an OR of 3.3 between need of oxytocin (*n* = 188) and labor induction, and an OR of 11.1 between early epidural anesthesia (*n* = 15) and labor induction.

**Table 3:** Maternal and neonatal outcomes

	Caesarean delivery		Vaginal delivery		p-value
	*n* = 169		*n* = 263		
	n	%	n	%	
Maternal outcomes					
Prophylactic antibiotic	154	91.1	87	33.1	<0.001
Oxytocin administration	158	93.5	244	92.8	0.78
For induction of labour	103	60.9	85	32.2	<0.001
Epidural/spinal analgesia	129	76.3	241	91.6	<0.001
Received before start of regular contractions	13	7.7	2	0.8	<0.001
Foetal scalp blood sampling	73	43.2	62	23.6	<0.001
Postpartum haemorrhage ≥ 1000 ml	55	32.5	37	14.1	<0.001
Meconium-stained amniotic fluid	59	34.9	61	23.2	0.008
Maternal intrapartum infection	22	13	5	1.9	<0.001
Maternal postpartum infection	12	7.1	5	1.9	0.007
Endometritis	5		4		
Wound infection	5		1		
Unspecified fever	2		0		
Neonatal outcomes					
Gestational weeks at delivery, mean (SD)	41.1	(1.3)	41,1	(1.4)	0.83
Male	99	58.6	127	48.3	0.04
Birth weight, mean(SD)	3720	(543)	3530	(483)	<0.001
Macrosomia (>4500 g)	11	6.5	3	1.1	0.004
Apgar 1 min < 7	20	11.8	20	7.6	0.13
Apgar 5 min < 7^a^	11	6.5	6	2.3	0.023
Umbilical artery pH ≤ 7.05^b^	1	0.6	9	3.6	0.06
Umbilical artery Base excess^b^	2	1.3	7	2.8	0.30
Neonatal infection	28	16.6	10	3.8	0.08
Suspected sepsis	15	53.6	6	60	0.001
Clinical sepsis	11	39.3	1	10	<0.001
Unspecified infection	2	1.2	3	30	0.85
Admission to neonatal intensive care unit	4	2.4	5	1.9	0.74
Admission to neonatal ward	43	25.4	24	9.1	<0.001

^a^Missing values 4

^b^Missing values 23

In univariate analysis, women who underwent caesarean section were slightly older (≥37 years) (*p* = 0.05), more obese (≥30 kg/m^2^) (*p* = 0.029), more often had gestational diabetes (*p* = 0.01), more often had a Bishop score ≤ 3 at start of IOL (*p* = 0.02), were more likely to need oxytocin for labour induction (*p* = < 0.001) and were more likely to have early epidural analgesia (*p* = 0.002) than women with vaginal delivery (Table 4). After multivariate logistic regression, only the associations with oxytocin induction and early epidural analgesia remained significant [OR 2.9 (95 % CI 1.8-4.5); *p* < 0.001 and OR 9.9 (95 % CI 2.1-47.5); *p* = 0.004]. Immediate or delayed timing of amniotomy or oxytocin administration were not associated with mode of delivery (Table 4). The indications for caesarean section are shown in Table 5. The median time from induction to birth was longer in women with caesarean delivery than in women with vaginal delivery (2090 min [range 170–4930] vs. 1706 min [range 445–4285]; *p* = 0.001).

**Table 4:** Univariate analysis for caesarean delivery, maternal intrapartum, postpartum and neonatal infections

		Caesarean delivery (n=169)^c^		Maternal intrapartum infection (n=27)^d^		Maternal postpartum infection (n=17)		Neonatal infection (n=38)		Neonatal clinical sepsis (n=12)^e^	
	n	OR	(CL 95 %)	OR	(CL 95 %)	OR	(CL 95 %)	OR	(CL 95 %)	OR	(CL 95 %)
Maternal age ≥ 37 (years)	43	1.9	1.0-3.6	0.7	0.2-3.1	1.8	0.2-13.9	1.1	0.4-3.2	4.9	1.4-17.0
IVF^a^	19	1.4	0.6-3.6	0	0	0.7	0.1-5.8	2	0.6-7.3	0	0
Smoking	61	1	0.6-1.8	1.8	0.7-4.7	1.2	0.3-5.6	1.2	0.5-2.9	2.1	0.5-7.9
BMI ≥ 30 kg/m^2^ (kg/m^2^)^b^	64	1.8	1.1-3.1	1	0.3-3.0	0	0	1.6	0.7-3.7	2	0.5-7.5
Gestational diabetes	81	1.9	1.2-3.1	3.3	1.5-7.4	3.8	0.5-29.2	1.4	0.6-3.1	4.6	1.4-14.6
Pre-eclampsia or hypertension	68	1	0.6-1.8	0.9	0.3-2.8	0		1.5	0.6-3.4	1.5	0.8-9.5
Bishop^a^ ≤ 3 at the start of induction	206	1.6	1.1-2.4	1.4	0.6-3.0	1.7	0.6-4.7	0.9	0.5-1.8	1.5	0.5-4.8
Cervical effacement > 2 cm at the time of amniotomy	32	0.8-3.4	0.6-2.1	1.1	0.2-4.7	1.5	0.3-7.0	0		0	0
Cervical dilation ≤ 3 cm at the time of amniotomy	19	1.6	0.8-3.4	0.7	0.2-3.0	0.9	0.1-7.7	0.99	0.3-3.0	1.2	0.2-10.0
Time of amniotomy after balloon expulsion											
≤2 h vs. >2 h	195	1.4	0.9-2.0	0.9	0.4-2.0	1.6	0.6-4.4	1	0.5-2.1	1.3	0.4-4.3
≤12 h vs.>12 h	47	0.9	0.5-1.7	2.5	0.9-6.5	2	0.3-15.6	0.8	0.3-2.2	1.7	0.4-8.2
Oxytocin for induction of labor	188	3.3	2.2-4.9	1	0.5-2.3	1.1	0.4-3.0	1.2	0.6-2.4	1.6	0.5-5.3
Time of starting oxytocin for IOL after amniotomy											
≤3 h vs. > 3 h	103	1.1	0.6-1.9	1.8	0.5-5.8	0.5	0.1-2.5	0.8	0.3-2.4	0.4	0.4-3.9
≤12 h vs. > 12 h	52	1.3	0.7-2.5	2	0.4-9.5			1.8	0.6-5.4	2.7	0.4-19.4
Early epidural analgesia	15	11.2	2.5-50.5	1	0.5-2.3	0.5	0.1-4.1	2.7	0.7-10.0	6	1.2-30.3

IVF; in vitro fertilization, BMI; body mass index

^a^Missing values 2

^b^Missing values 4

^c^Maternal age ≥ 37 (p=0.045), BMI≥ 30 (p=0.029), gestational diabetes (p=0.01), Bishop ≤ 3 (p=0.018), oxytocin induction (p<0.001), early epidural analgesia (p=0.002)

^d^Gestational diabetes (p=0.004)

^e^Maternal age ≥ 37 (p=0.013), gestational diabetes (p=0.01), early epidural analgesia (p=0.029)

**Table 5:** Main indication for caesarean delivery (*n =* 169)

	n	Percent
Foetal distress	63	37.3
Infection	12	7.1
Failure to progress	85	50.3
Labour arrest	53	31.4
Failed induction	32	18.9
Bleeding	1	0.6
Pre-eclampsia	3	1.8
Umbilical cord prolapse	1	0.6
Maternal exhaustion	2	1.2
Failed vaginal instrumental delivery	2	1.2

The maternal intrapartum infection rate was 6.3 % (*n* = 27), and the postpartum infection rate was 3.9 % (*n* = 17) (Table 3). Both intrapartum and postpartum infections were more common among women who had caesarean sections than women who delivered vaginally [OR 7.7 (95 % CI 2.9-20.8); *p* < 0.001 and OR 3.9 (95 % CI 1.4-11.4); *p* = 0.007]. Overall 2.8 % (*n* = 12) of the women had caesarean sections due to suspected intrapartum infection. Two of these women had blood culture-positive sepsis. In univariate analysis, gestational diabetes was associated with increased risk of intrapartum infection (*p* = 0.004) (Table 4) and this association remained after multivariate regression analysis [OR 4.3 (95 % CI 1.7-11.0); *p* = 0.002].

Neonatal outcomes are shown in Table 3. The overall neonatal infection rate was 8.8 % (*n* = 38). No cases of blood culture-positive neonatal sepsis were found. Both clinical and suspected sepsis were more common in neonates delivered by caesarean section than in those delivered vaginally [OR 5.0 (95 % CI 2.4-10.6); *p* < 0.001 and OR 18.2 (95 % CI 2.3-142.6); *p* = 0.001]. By univariate analysis, maternal age ≥ 37 (*p* = 0.01), gestational diabetes (*p* = 0.01) and early epidural analgesia (*p* = 0.029) were associated with neonatal clinical sepsis (Table 4). By multivariate regression analysis, the significance remained only for early epidural analgesia [OR 10.5 (95 % CI 1.4-76); *p* = 0.02].

## Discussion

We found that the need for oxytocin for induction and early epidural analgesia were associated with caesarean delivery in nulliparous women undergoing IOL by Foley catheter. Gestational diabetes was associated with maternal intrapartum infection, while early epidural analgesia was associated with neonatal infection. Surprisingly, Bishop score at the start of IOL, duration of the balloon remaining in the cervical canal, timing of amniotomy or timing of oxytocin induction had no association with caesarean section or infections. We acknowledge the limitations of our retrospective single-institution study; we may have been susceptible to selection bias, and our results may not be applicable to other settings.

The caesarean section rate in our study was 39.1 %. Frederiks et al. found a similar 42 % caesarean delivery rate in nulliparous women with a variety of induction methods [8]. In another recent study, the overall rate of successful IOL in combination with different methods and an unfavourable Bishop score (<6) at 41 weeks of gestation was 51.3 % [7]. We found a higher rate of successful labour induction resulting in vaginal delivery than these other recent studies. (The caesarean section rate among the 59 excluded women with sequential use of Foley catheter and intravaginal prostaglandin was even higher, 45.8 % (*n* = 27)).

The degree of cervical ripeness in this study was assessed using the Bishop score, which was originally derived from observations made on multiparous women [12]. We used Bishop score ≥ 6 as a marker for a ripened cervix, at which time amniotomy and oxytocin augmentation may be used. Perhaps a more modern concept for a favourable cervix in a nulliparous women would be Bishop score ≥ 8 [13].

In previous studies, low Bishop score (<5), increased maternal age, obesity and large birth weight have been associated with induction failure and operative delivery [4, 5, 8, 14–17]. This trend was also seen in our study. However, in multivariate regression analysis, only the need for oxytocin to induce contractions and early epidural analgesia remained associated with caesarean delivery, as also seen in a previous study [18]. It has been suggested that since IOL, oxytocin use, low parity, prelabour rupture of membranes and obesity are all linked with increased caesarean delivery risk [19], this may explain the increased caesarean rate related to early epidural [20]. However, the request for analgesia early in labour (cervical dilation of ≤ 3 cm) may be a marker for other risk factors for caesarean delivery [21].

The most common indication for caesarean delivery was failure to progress (failed induction and labour arrest), as was also noted in previous studies [3, 5]. Several studies suggest that a substantial proportion of women undergoing IOL and remaining in the latent phase for 12–18 hours with oxytocin administration and ruptured membranes will deliver vaginally if induction is continued [22–24]. Previous studies have shown that active labour does not occur until about 6 cm of cervical dilation in women undergoing IOL, and that a diagnosis of failed induction should be made with caution prior to that stage [25, 26]. ACOG clinical guidelines recommend that arrest in the first stage of labour should be reserved for women ≥ 6 cm of cervical dilation with ruptured membranes who are failing to progress despite of 4 hours of adequate uterine activity or 6 hours of oxytocin administration without cervical change [11]. In our study, the definitions for failed induction and arrest of labour varied. This suggests that some women undergoing IOL in our clinic might have been diagnosed with failed induction or labour arrest too early.

Macones et al. demonstrated that early amniotomy at ≤ 4 cm of cervical dilation during IOL shortened the time to delivery in nulliparous women, but did not impact the caesarean delivery rate [27], which was in line with our results. Oxytocin is more often used with Foley catheter IOL compared to pharmaceutical IOL or spontaneous labour [3]. Oxytocin use was high in our study, which may partly explain our high caesarean section rate. The timing of oxytocin administration for IOL was not associated with caesarean section or maternal or neonatal infections. To our knowledge, no other study has focused on this in the context of Foley catheter IOL.

In one previous study, Foley catheter IOL was associated with increased infectious morbidity [28]. In several other studies, as well as in a recent Cochrane review, Foley catheter IOL has not been linked to increased maternal or neonatal infection rates [3, 29, 30]. This is in agreement with our results. In our study, gestational diabetes was associated with maternal intrapartum infection. In a previous Danish study, type II or gestational diabetes showed a modestly increased risk of post-caesarean infection [31]. In our study, the rate of neonatal clinical sepsis was similar to that in previous studies [3, 29], but the rate of suspected neonatal infections was higher. We believe that potential bias related to Foley catheter induction and longer induction-to-delivery interval resulted in more neonates being admitted to the neonatal ward for observation or antibiotic administration. The duration of labour after ruptured membranes has previously been linked to neonatal infections [9, 10]. In our study, early epidural analgesia was associated with caesarean delivery and neonatal infections. This can be explained by the prolongation of labour.

## Conclusions

Multivariate analysis found that early epidural analgesia and the need for oxytocin to induce contractions were associated with caesarean section in nulliparous women undergoing Foley catheter IOL. Gestational diabetes and early epidural analgesia were associated with infectious morbidity. These data are important when optimising the management of induced labour. The decision to induce labour should be carefully considered since the process has risks associated with it.

## Abbreviations

IOL: Induction of labour
GBS: Streptococcus agalactie B
OR: Odds ratio
CI: Confidence interval
IVF: In vitro fertilisation
BMI: Body mass index
